# Supplementary material for: Cancer-specific PERK signaling drives invasion and metastasis through CREB3L1
Source: Nat Commun. 2017 Oct 20;8:1079. doi: 10.1038/s41467-017-01052-y (PMC5651903; doi:10.1038/s41467-017-01052-y)
Supplement: Supplementary file 1 — Supplementary Information [file 41467_2017_1052_MOESM1_ESM.pdf]

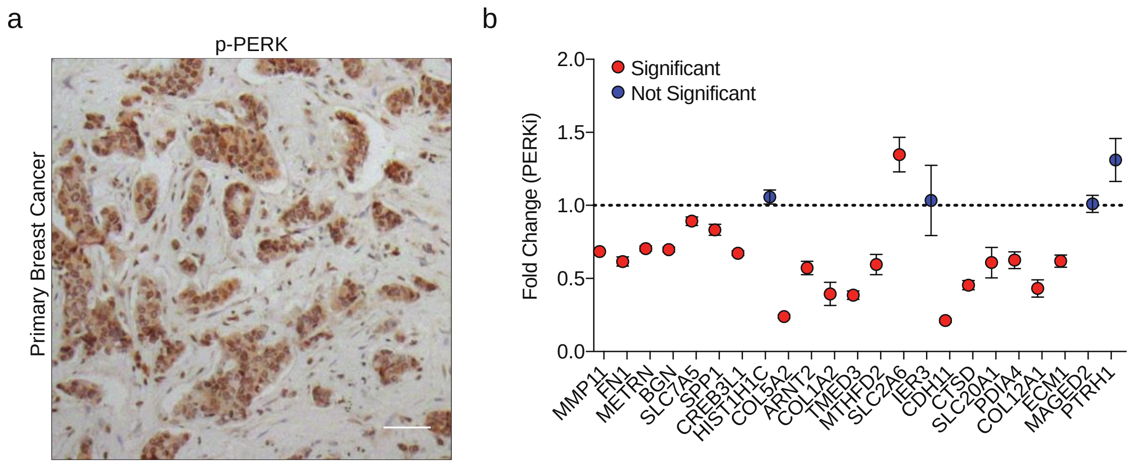

Supplementary Figure 1. PERK is phosphorylated in primary breast cancer and induces the expression of cancer-specific PERK genes

(a) IHC staining for phosphorylated PERK in a set of 50 breast cancer samples (Biomax, #BR10010d). Phosphorylation of PERK was found in 35 out of 50 primary breast cancer tissue samples. Shown was a representative image of staining (scale bar: 40 μm). **(b)** qPCR showing the fold change of the expression of 23 cancer-specific PERK genes in SUM159 cells treated with GSK2656157, a PERK inhibitor, relative to vehicle control-treated cells, n=3. Data are represented as mean ± SEM. Red dot indicates $p<0.05$ (Student's *t*-test).

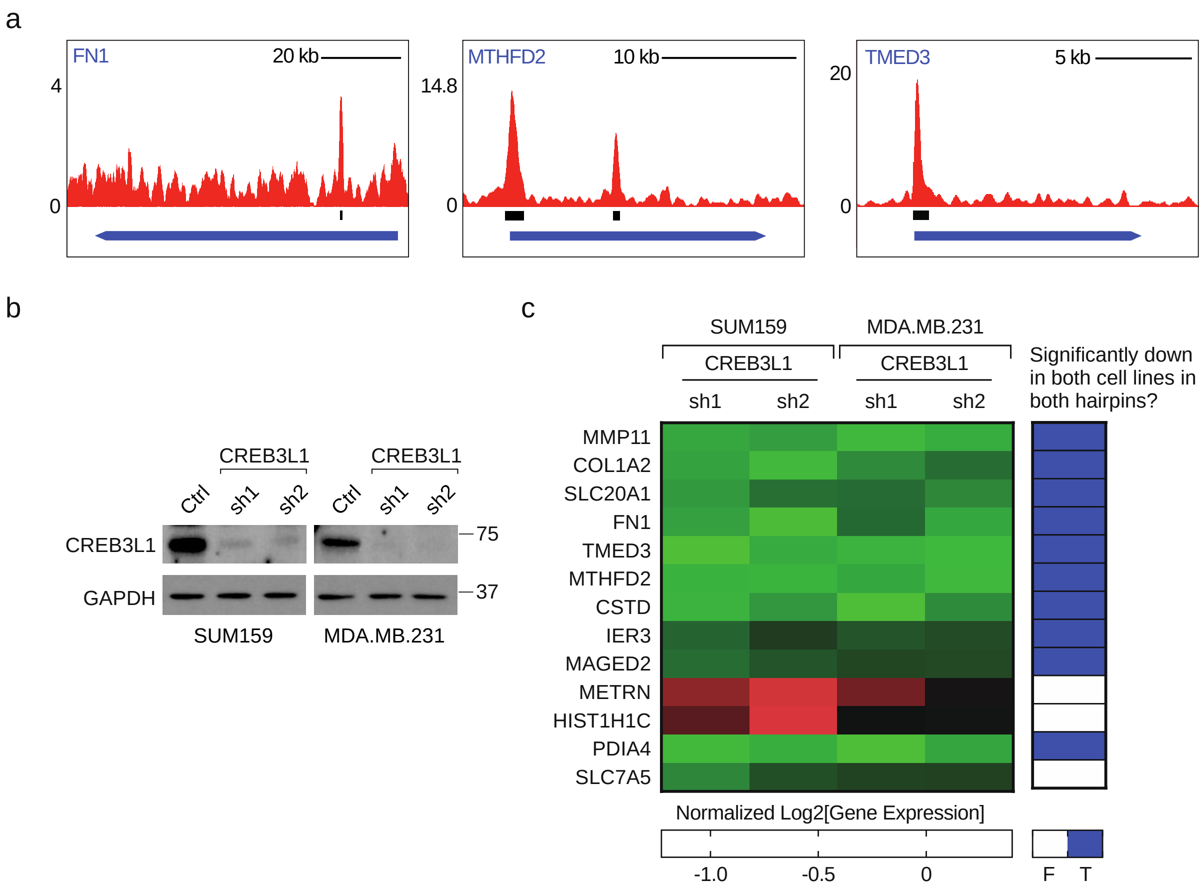

Supplementary Figure 2. CREB3L1 regulates the expression of Cancer-Specific PERK genes

(a) Distribution of CREB3L1 ChIP-seq fold-enrichment over input control at the locus of three CSPS genes, FN1, MTHFD2, and TMED3. **(b)** Western blot showing the expression of CREB3L1 in SUM159 and MDA.MB.231 cells transduced with shRNAs targeting luciferase or CREB3L1. **(c)** Heatmap showing the fold change of the expression of 13 putative CREB3L1-target genes in SUM159 and MDA.MB.231 cells transduced with shRNAs targeting CREB3L1, relative to control shRNA-transduced cells, n=4. Genes that were significantly down-regulated by both shRNAs in both cell lines were marked in blue.

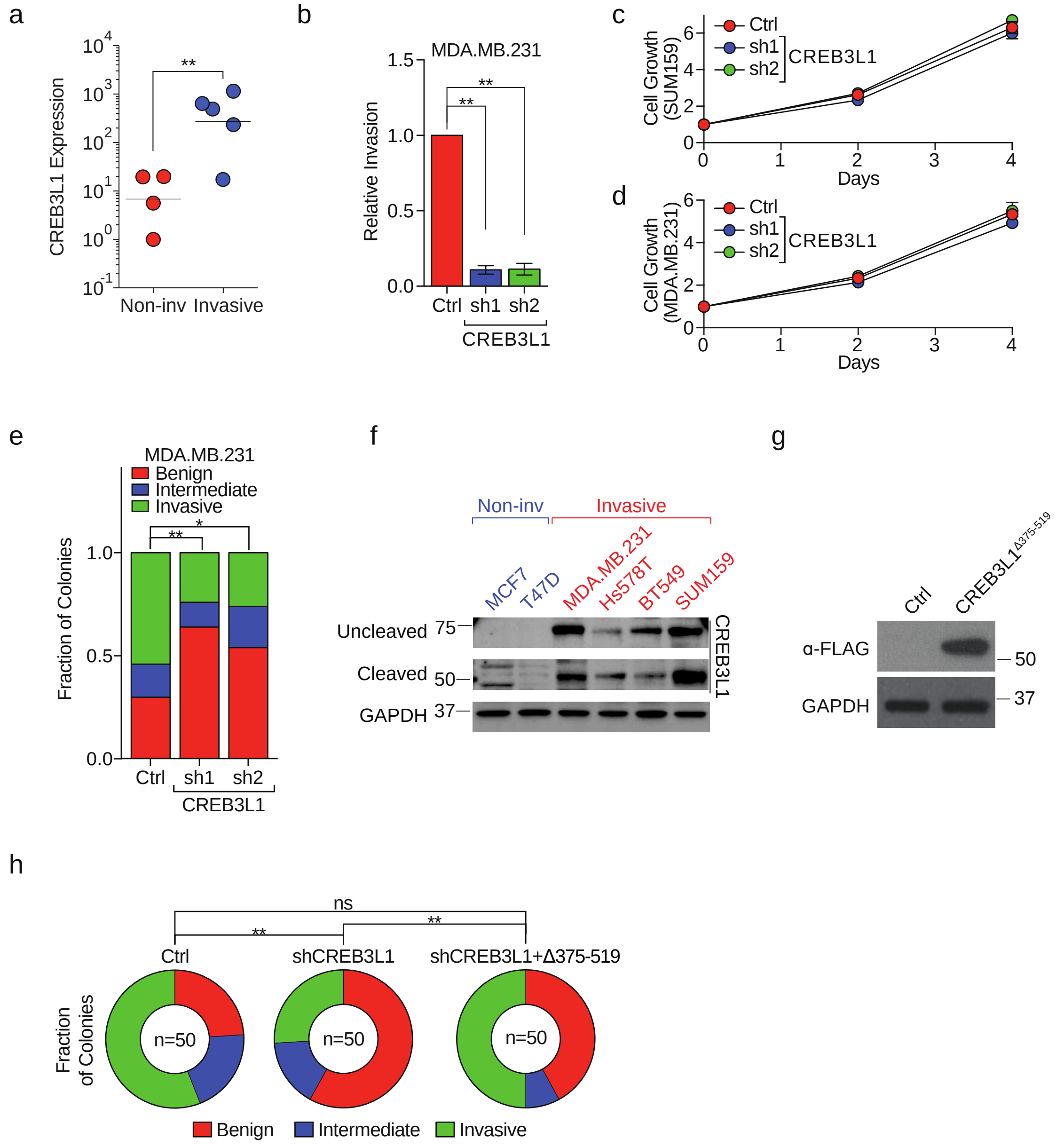

Supplementary Figure 3. CREB3L1 is up-regulated in invasive breast cancer cell lines and required for cell invasion

(a) qPCR showing the relative expression of CREB3L1 in 4 luminal human breast cancer cells, MCF7, T47D, BT474, and ZR-75-3, and 5 basal-B cell lines, SUM159, MDA.MB.231, MDA.MB.157, BT549, and Hs578T, n=3. Data are represented as mean ± SEM. * indicates $p<0.05$; **indicates $p<0.01$ (Student's *t*-test). **(b)** Quantification of cell invasion of

MDA.MB.231 cells transduced with shRNAs targeting luciferase or CREB3L1 in a basement membrane-coated trans-well assay, n=3. Data are represented as mean ± SEM. * indicates p<0.05; **indicates p<0.01 (Student's *t*-test). **(c)** and **(d)** Quantification of cell growth of SUM159 cells (c) or MDA.MB.231 cells (d) transduced with shRNAs targeting luciferase or CREB3L1, n=3. **(e)** Quantification of colony types formed by MDA.MB.231 cells transduced with shRNAs targeting luciferase or CREB3L1 in a seven day 3D matrigel invasion assay, n=50. p=0.0022 for sh1 and p=0.0139 for sh2, Chi-square test. **(f)** Western blot showing the uncleaved and cleaved CREB3L1 in MCF7, T47D, SUM159, MDA.MB.231, BT549, and Hs578T cells. **(g)** Western blot showing the expression of truncated CREB3L1 in HMLE cells overexpressing flag tagged CREB3L1$^{\Delta 375\text{-}519}$. **(h)** Quantification of colony types formed by MDA.MB.231 cells transduced with an shRNA targeting luciferase, an shRNA targeting CREB3L1, or an shRNA targeting CREB3L1 and a construct expressing flag tagged CREB3L1$^{\Delta 375\text{-}519}$ in a seven day 3D matrigel invasion assay, n=50. p=0.0017 comparing Ctrl vs shCREB3L1, p=0.07 comparing Ctrl vs shCREB3L1+ Δ 375-519, and p=0.04 comparing shCREB3L1 vs shCREB3L1+ Δ 375-519, Chi-square test.

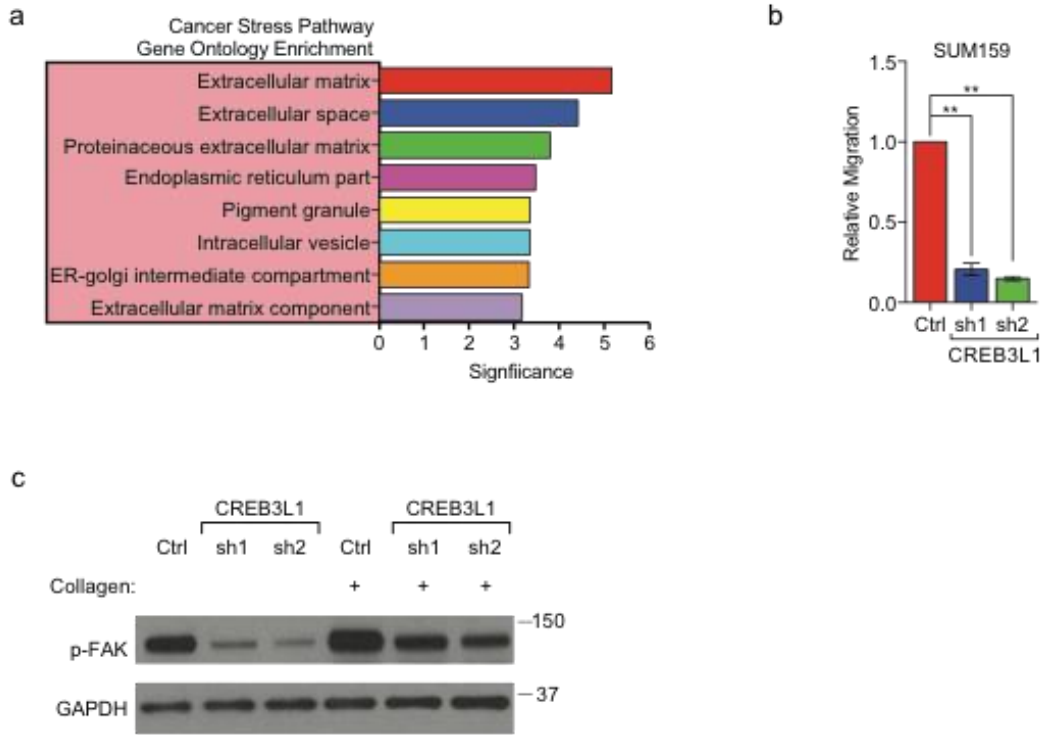

Supplementary Figure 4. CREB3L1 regulates ECM gene expression and activates FAK

(a) Gene ontology analysis of cancer-specific PERK set regulated by CREB3L1 using cellular components set provided by the molecular signature database. Significance is represented as the negative $\log_{10}$ of the p-value. **(b)** Quantification of cell migration of SUM159 cells transduced with shRNAs targeting luciferase or CREB3L1 in a trans-well assay (not Matrigel-coated), n=3. **(c)** SUM159 cells transduced with shRNAs targeting luciferase or CREB3L1 were cultured in dishes coated with or without type I collagen for 24 hours prior to protein extraction and western blotting. The expression of phosphorylated FAK and GAPDH were examined by western blot. Data are represented as mean ± SEM. * indicates $p<0.05$; **indicates $p<0.01$ (Student's *t*-test).

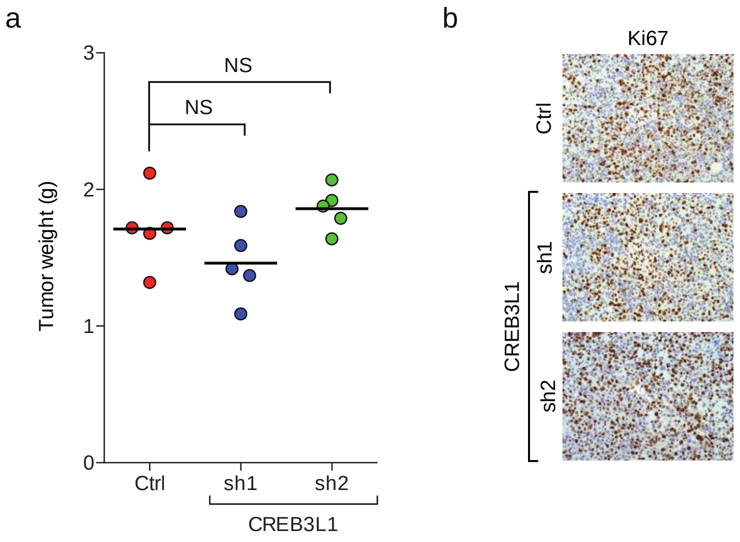

Supplementary Figure 5. Knockdown of CREB3L1 does not affect tumor growth *in vivo*

(a) Tumor weights from mice that were inoculated with MDA.MB.231-LM2 cells transduced with a control hairpin or two hairpins targeting CREB3L1. Lines represent the mean, n=5. **(b)** IHC for Ki67 expression on tumors sections collected from the mice in (a).

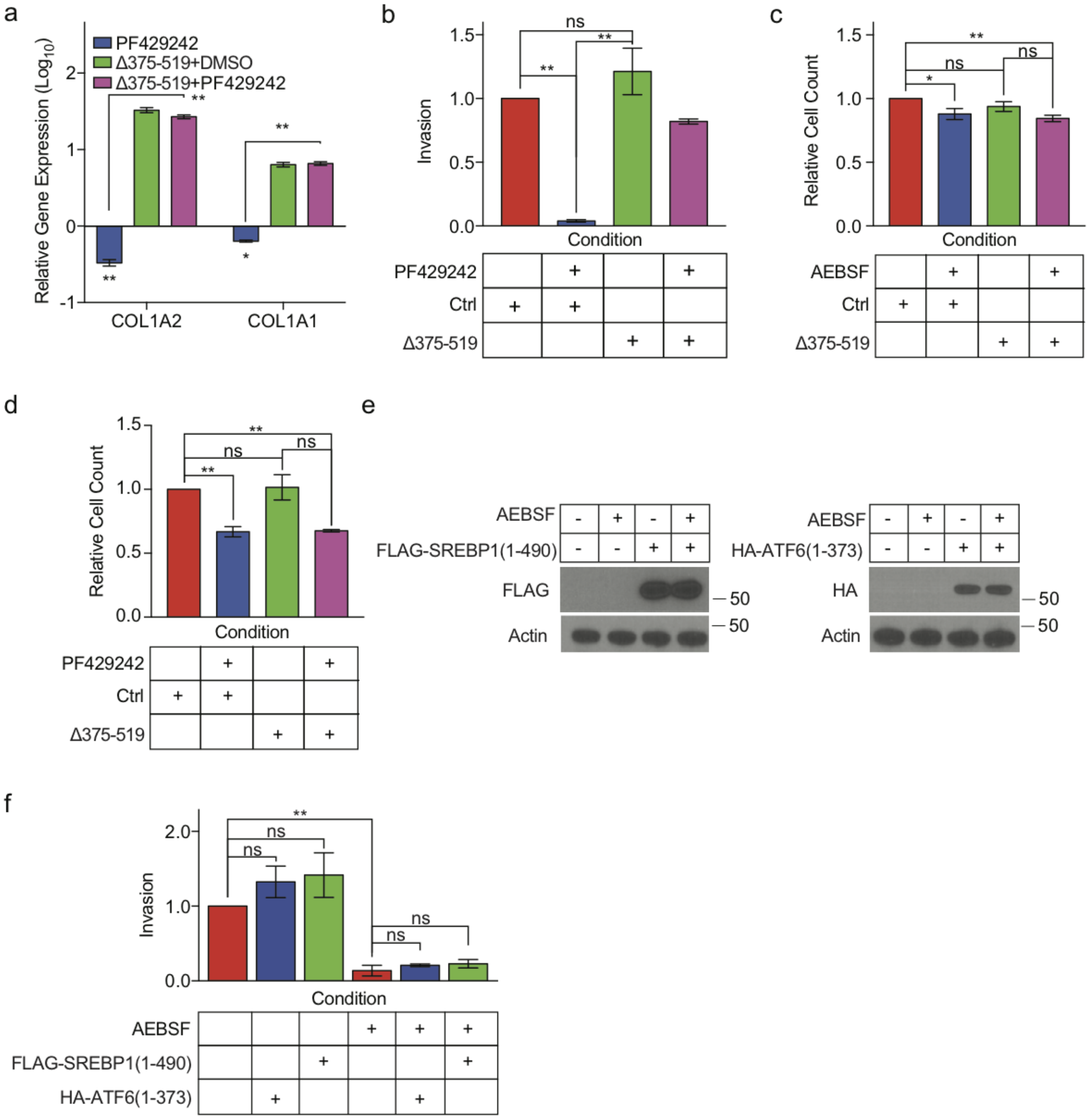

Supplementary Figure 6. Suppressing CREB3L1 with chemical inhibitors of proteases in vitro

(a) SUM159 cells were treated with solvent control or PF429292, in combination of transduction with control or CREB3L1$^{\Delta 375\text{-}519}$. qPCR analyses were performed to quantify the expression of ECM genes *COL1A1* and *COL1A2*. The gene expression was normalized to the solvent treated

control transduction samples, n=3. **(b)** Quantification of cell invasion of cells from (a) in a basement membrane-coated trans-well assay, n=3. **(c)** 16 hours after SUM159 cells were treated with solvent control or AEBSF, in combination of transduction with control or CREB3L1$^{\Delta 375\text{-}519}$, cell number was counted and plotted for cell survival, n=3. **(d)** 16 hours after SUM159 cells were treated with solvent control or PF429242, in combination of transduction with control or CREB3L1$^{\Delta 375\text{-}519}$, cell number was counted and plotted for cell survival, n=3. **(e)** Western blot showing expression of FLAG tagged SREBP1 (N terminus 1-490) or HA tagged ATF6 (N terminus 1-373) in SUM159 cells treated with the indicated condition. **(f)** Quantification of cell invasion of SUM159 cells treated with solvent control or AEBSF, in combination of transduction with control construct, FLAG tagged SREBP1(N terminus 1-490), or HA tagged ATF6 (N terminus 1-373), in a basement membrane-coated transwell assay, n=3. Data are represented as mean ± SEM. * indicates $p<0.05$; **indicates $p<0.01$ (Student's *t*-test).

a

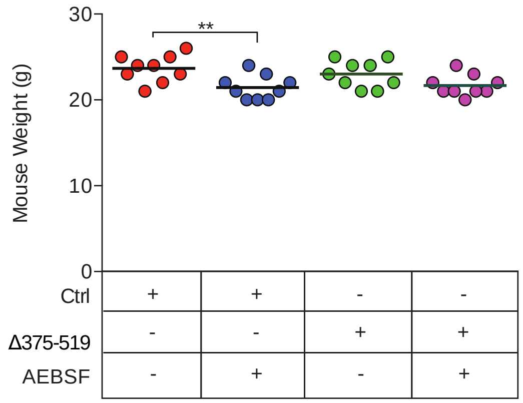

b

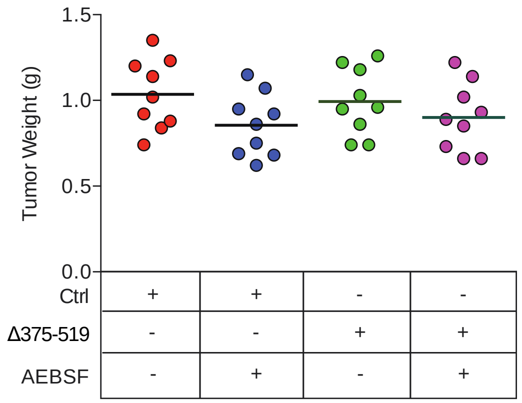

Supplementary Figure 7. Effect of chemical inhibition of CREB3L1 on the growth of mouse or the growth of primary tumors

(a) Mouse weights following treatment with AEBSF or solvent control. Mice were inoculated with MDA.MB.231-Luc-LM2 cells transduced with a control plasmid or a constitutively active CREB3L1 (CREB3L1 $^{\Delta375\text{-}519}$), n=9. **(b)** Tumor weights from the conditions described in (a), n=9. * indicates $p<0.05$; **indicates $p<0.01$ (Student's *t*-test).

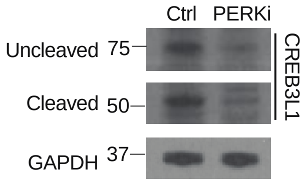

Supplementary Figure 8. Inhibition of PERK reduces the expression of CREB3L1 but does not change its proteolytic cleavage

Western blot showing the expression of uncleaved and cleaved CREB3L1 in SUM159 cells treated with vehicle control or a PERK inhibitor, GSK2656157.

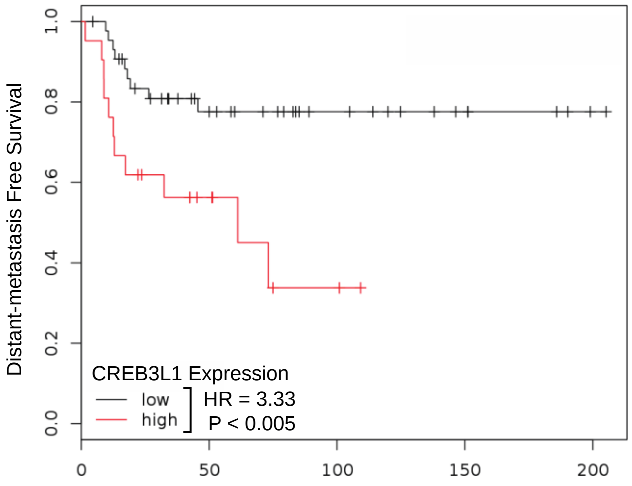

Supplementary Figure 9. Expression of CREB3L1 predicts distant metastasis-free survival in the mesenchymal subtype of triple-negative breast cancers

Kaplan-Meier curves showing significant association of elevated CREB3L1 expression (red line) with shorter distant metastasis-free survival in a cohort of patients with triple-negative breast cancer of the mesenchymal subtype (KMplotter, mesenchymal subtype of TNBC).

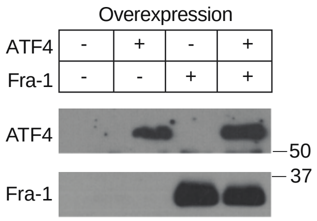

Supplementary Figure 10. Overexpression of ATF4 and Fra-1 in HMLE cells

Western blot showing the expression of ATF4 and Fra-1 in HMLE cells overexpressing a control construct, ATF4, Fra-1, or a combination of ATF4 and Fra-1.

Figure 2h

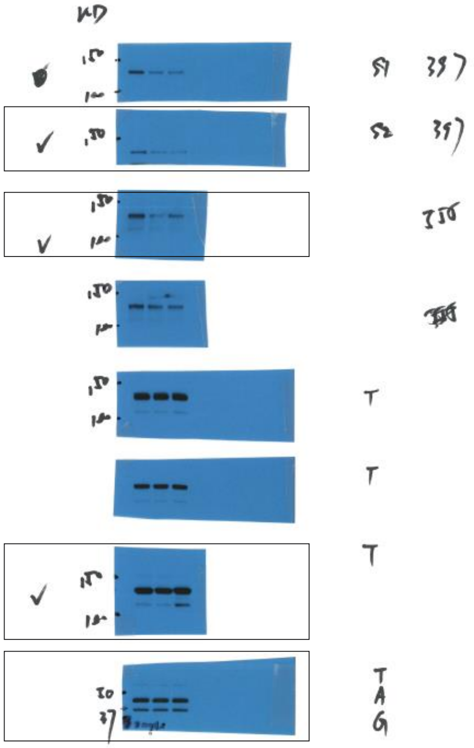

Figure 4a

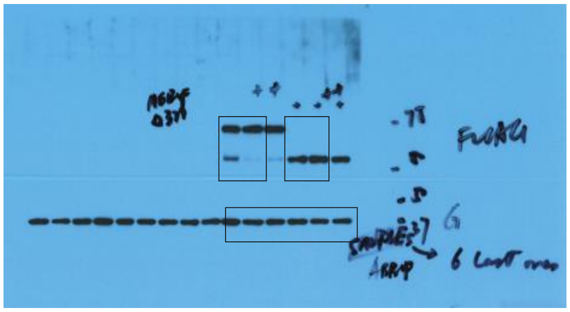

Figure 4a

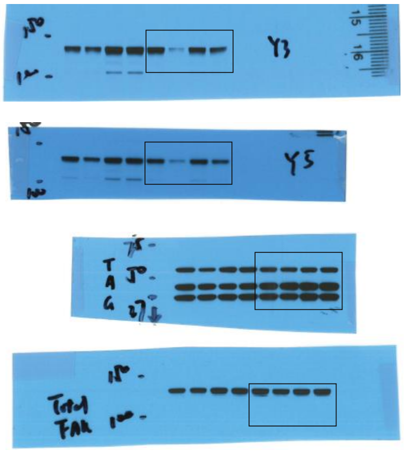

Figure 5f

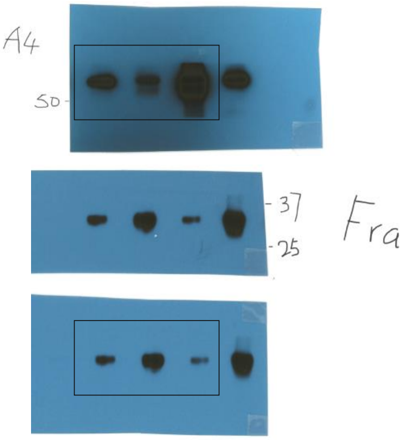

Supplementary Figure 2b

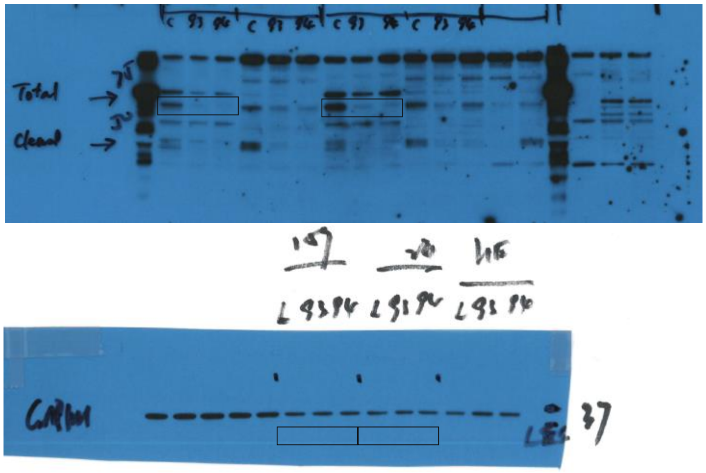

Supplementary Figure 3f

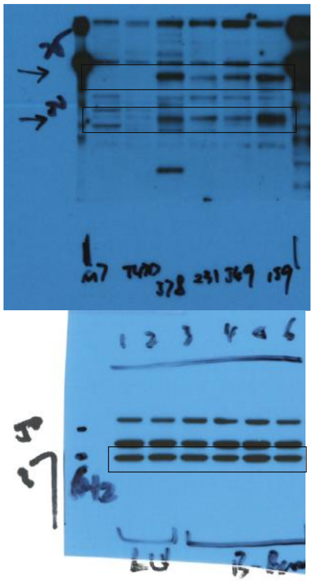

Supplementary Figure 4c

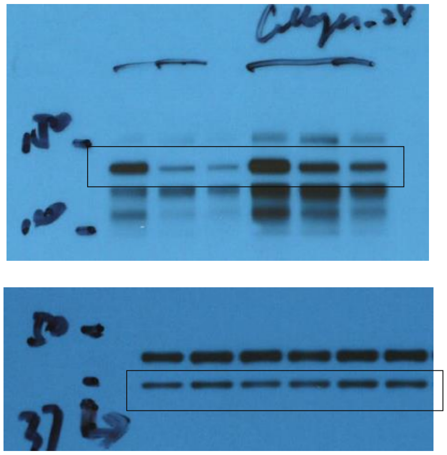

Supplementary Figure 6e

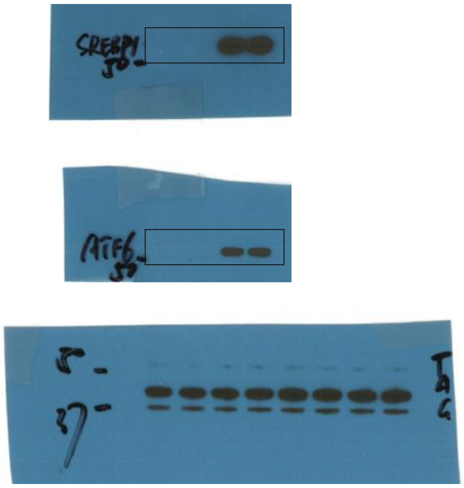

Supplementary Figure 8

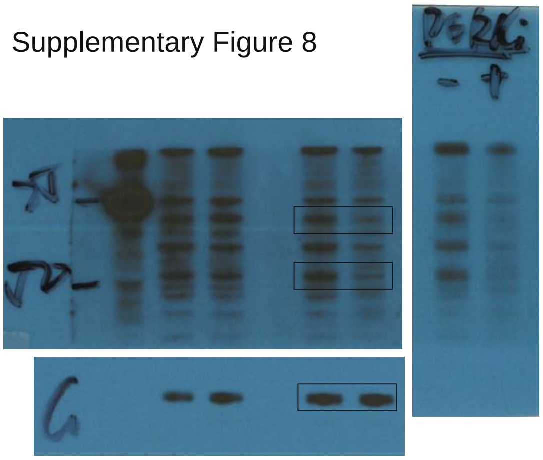

Supplementary Figure 10

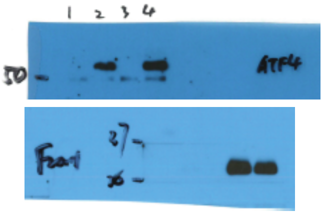

Supplementary Figure 11. Uncropped gel images of western blots

Uncropped gel images of western blots showed in main figures and supplementary figures.
