# Supplementary material for: Cancer-specific PERK signaling drives invasion and metastasis through CREB3L1
Source: Nat Commun. 2017 Oct 20;8:1079. doi: 10.1038/s41467-017-01052-y (PMC5651903; doi:10.1038/s41467-017-01052-y)
Supplement: Supplementary file 3 — Description of Additional Supplementary Files [file 41467_2017_1052_MOESM3_ESM.pdf]

Description of Additional Supplementary Files

File Name: Supplementary Data 1
Description: A list of ~400 genes up-regulated by PERK signaling according to a previous report (Reference #20 of the revised manuscript).
